# Short-term preliminary clinical results from HIPOCRAT-ESK study: a cohort, non-interventional, prospective, real-world study of functional remission in TRD patients treated with Esketamina nasal spray.

**DOI:** 10.1192/j.eurpsy.2026.11420

**Published:** 2026-07-31

**Authors:** T. Caraballo Lopez, M. García Dorado, F. Mora, J. M. Crespo Iglesias, Á. Ibáñez Cuadrado, N. Cardoner Álvarez

**Affiliations:** 1 Johnson & Johnson Spain; 2 Psychiatry, Hospital Infanta Leonor, Madrid; 3 Hospital Marítimo de Oza, A Coruña; 4 Hospital Ramón y Cajal, Madrid; 5 Hospital Santa Creu i Sant Pau, Barcelona, Spain

## Abstract

**Introduction:**

Approximately up to 55% of patients diagnosed with major depressive disorder (MDD) meet treatment resistant depression (TRD) criteria 1, characterized by a worsened prognosis2,3. In this context, esketamina nasal spray (ESK NS) arises as a novel therapeutic option with a different mechanism of action, based in the regulation of the glutamatergic pathway, directed to enhance brain neuroplasticity4.

**Objectives:**

To report the initial results of HIPOCRAT-ESK, the first real-world, prospective study evaluating functional remission of TRD patients treated with ESK NS based in Spanish clinical practice.

**Methods:**

HIPOCRAT-ESK is a cohort, non-interventional, prospective, real-world study evaluating functions remission as a primary endpoint in TRD patients treated with ESK NS as per the Spanish clinical practice. Total sample consists in 300 TRD patients with a prospective follow-up of 12 months. It evaluates clinical evolutions using MADRS, SDS, PHQ-9 and EQ-5D-5L scales. We present the short-term preliminary results (first 25% of the total sample recruited) focusing on clinical evolution.Basal sociodemographic and safety profile is included in the analysis.

**Results:**

83 patients were included in the intention to treat analysis and 95 in the safety analysis. The sample was severely depressed, with a basal MADRS total score of 37 points. Presence of TRD and psychiatric comorbidities was high, especially anxiety disorder and borderline personality disorder (10.84% each). Real-world use of ESK NS achieved positive short -term clinical results. Patients achieved mean clinically meaningful improvement in MADRS mean total score, as soon as in the second treatment administration, with sustained improvements during the induction phase (mean total MADRS score decrease of 12.55 points vs basal). Clinical response and clinical remission were achieved by 21.43% and 7.14% of patients, respectively, by the end of induction phase. Patients in HIPOCRAT-ESK were included in different TRD stages based in the number of prior failed AD therapies. The analysis showed that, therapeutic inertia and treatment chronicity decreases the rates of clinical and remission response (Table 3). Key safety findings were aligned with already published data for ESK NS.

**Image 1:**
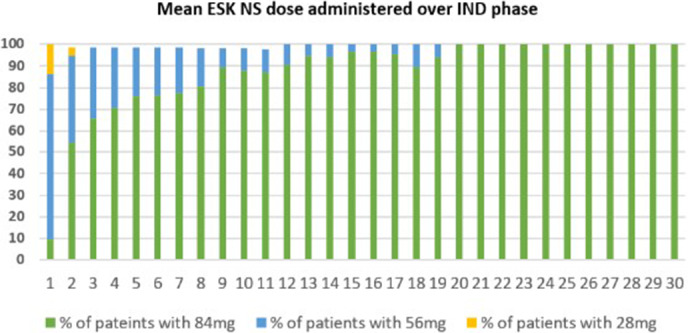
Image 1: Long description.

**Conclusions:**

HIPOCRAT-ESK is the first prospective, real-world evidence study aiming to evaluate functional remission in TRD patients treated with ESK NS as primary endpoint. Preliminary short-term analysis shows promising efficacy results, with clinical improvements observed as soon as second administration session and sustained over the induction phase

**Disclosure of Interest:**

T. Caraballo Lopez Employee of: Full-time employee, M. García Dorado Employee of: Full-time employee, F. Mora Grant / Research support from: Research support, Consultant of: Consultant for Johnson & Johnson, J. M. Crespo Iglesias Grant / Research support from: Research support recieved form Johnson & Johnson, Consultant of: Act as a consultant for Johnson & Johnson, Á. Ibáñez Cuadrado Grant / Research support from: Recieved research support from Johnson & Johnson, Consultant of: Acts as a consultant for Johnson and Johnson, N. Cardoner Álvarez Grant / Research support from: Recieves research support from Johnson & JOhnson, Consultant of: Acts as a consultant for Johnson & Johnson

## Long descriptions

### Image 1: Long description

A stacked bar chart titled Mean E S K N S dose administered over I N D phase. The y-axis represents the percentage of patients from 0 to 100. The x-axis lists 30 sequential time points labeled 1 through 30. A legend at the bottom identifies three dose categories: green for % of patients with 84 m g, blue for % of patients with 56 m g, and yellow for % of patients with 28 m g.

* At time point 1, the 84 m g dose (green) is at approximately 10%, the 56 m g dose (blue) is approximately 75%, and the 28 m g dose (yellow) is approximately 15%.

* From time points 2 to 11, there is a steady linear increase in the green 84 m g segment, rising from roughly 55% to 88%, while the blue 56 m g segment decreases and the yellow 28 m g segment disappears after time point 2.

* From time points 12 to 19, the green 84 m g segment dominates at over 90%, with small blue 56 m g segments remaining at the top of the bars.

* From time points 20 to 30, the bars are entirely green, indicating 100% of patients received the 84 m g dose.

Navigate back to Image 1:.

